# Effects of Outdoor Rearing System on the Growth Performance and Blood Parameters of Duroc Pigs

**DOI:** 10.3390/ani16071040

**Published:** 2026-03-28

**Authors:** Kaliyah Hayes, Andrea Gentry-Apple, Lin Yang, Julisa Cruz, Joseline Mora-Obrajero, Daisha Peele-Kendrick, Shilei Zhang, Derrick Coble, Yongjie Wang

**Affiliations:** 1 Department of Animal Science, College of Agriculture and Environmental Science, North Carolina Agricultural and Technical State University, Greensboro, NC 27411, USA; 2 College of Animal Science and Technology, Shihezi University, Shihezi 832003, China

**Keywords:** outdoor rearing, whole blood parameters, serum biochemical parameters, growth performance, Duroc pigs

## Abstract

The objective of this study was to evaluate the physiological mechanisms and health resilience of Duroc pigs reared in an outdoor system compared to a conventional indoor system. A total of 24 Duroc pigs (approximately 3 months of age) were randomly assigned to either an indoor (IN, *n* = 12) or an outdoor (OUT, *n* = 12) rearing system for a 45-day trial. Growth performance (body weight and ADG) and spleen organ index were not significantly different between the two rearing systems (*p* > 0.05). Hematological profiles, including leukocyte and erythrocyte indices, showed no significant differences (*p* > 0.05), although plateletcrit tended to decrease in the OUT group (*p* = 0.08). For serum biochemical parameters, pigs in the OUT group exhibited significantly higher concentrations of total protein, triglycerides, calcium, and sodium compared to those in the IN group (*p* < 0.05). Additionally, serum albumin and glucose levels tended to be higher in the OUT group (*p* < 0.01). No significant differences were observed in liver and muscle enzyme activities (AST, ALP, GGT, CK) between the treatments (*p* > 0.05). In conclusion, outdoor rearing did not compromise growth performance or induce chronic physiological stress or tissue damage in Duroc pigs. Instead, it promoted active energy and lipid mobilization, enhanced protein metabolism, and improved mineral homeostasis, demonstrating the robust physiological adaptability of the Duroc breed to outdoor environments.

## Simple Summary

Consumers increasingly prefer pork from farms that prioritize animal welfare, such as those that allow outdoor living. However, farmers are concerned that the challenges of outdoor living, such as variable weather and increased physical activity, may stress the pigs and reduce their growth. This study aimed to determine how raising Duroc pigs outdoors affects their growth and overall health compared with traditional indoor farming. Over 45 days, we compared the two groups by measuring their weight and testing their blood samples. We found that outdoor pigs grew as well as indoor pigs. Furthermore, the blood tests showed no signs of chronic stress or organ damage in the outdoor pigs. Instead, they had higher levels of protein, fat, and minerals, such as calcium, likely due to exposure to natural sunlight. In conclusion, raising Duroc pigs outdoors does not adversely affect their growth or health; rather, they adapt strongly and positively to the environment. This research is valuable to society because it demonstrates that farmers can adopt animal-friendly outdoor systems to raise healthy pigs, thereby satisfying public demand for ethically produced food without sacrificing farming efficiency.

## 1. Introduction

In modern swine production, there is a growing consumer and industry demand for rearing systems that prioritize animal welfare and environmental sustainability [1]. Consequently, outdoor and free-range rearing systems have gained significant attention as viable alternatives to conventional indoor confinement [2]. Outdoor rearing provides pigs with more space, complex environmental stimuli, and the freedom to express natural behaviors, which can significantly enhance their psychological well-being [3]. However, outdoor environments also expose pigs to continuous physiological challenges, including fluctuating ambient temperatures, increased physical activity, and greater potential exposure to soil-borne pathogens and parasites [4].

These environmental challenges have sparked a debate regarding the biological cost of outdoor rearing. A primary concern is that the increased energy expenditure required for thermoregulation and physical activity outdoors may divert essential nutrients away from muscle deposition, thereby negatively impacting growth performance and feed efficiency [5]. While some studies have reported reduced average daily gain (ADG) and poorer feed conversion ratios in outdoor-reared pigs [6], others have suggested that pigs can compensate for these demands through increased feed intake or enhanced metabolic efficiency without compromising growth [7]. Furthermore, the continuous exposure to environmental stressors raises questions about the systemic immune status and physiological homeostasis of outdoor-reared pigs.

To accurately evaluate the viability of outdoor rearing, it is crucial to investigate beyond basic growth metrics and examine the underlying physiological mechanisms of adaptation. Hematological profiles, such as the complete blood count (CBC), provide direct insights into the active cellular immune response and systemic inflammation [8]. Simultaneously, serum biochemical parameters—including total protein, liver and muscle enzymes, metabolites, and electrolytes—serve as highly sensitive biomarkers for evaluating nutritional status, energy metabolism, tissue integrity, and mineral homeostasis [9]. Together, these physiological indicators define the “health resilience” of the animal—its capacity to cope with and adapt to environmental stressors without suffering pathological damage.

The Duroc breed is widely recognized in the swine industry for its rapid growth rate, superior meat quality, and robust physical constitution [10]. However, comprehensive physiological profiles detailing how Duroc pigs adapt their immune and metabolic systems to outdoor environments remain limited. Therefore, this experiment was conducted to investigate the effect of an outdoor rearing system compared to a conventional indoor system on the growth performance, organ development, hematological profiles, and serum biochemical parameters of Duroc pigs. The objective of this study is to elucidate the physiological mechanisms underlying the adaptability of Duroc pigs to outdoor environments, providing scientific evidence to support welfare-friendly swine production.

## 2. Materials and Methods

### 2.1. Experimental Animals, Design, and Management

All experimental procedures involving animals were conducted in accordance with the Animal Experimental Guidelines provided by the Institutional Animal Care and Use Committee (IACUC) of North Carolina Agricultural and Technical State University. A total of 24 Duroc pigs (12 castrated males and 12 females), approximately 3 months of age, were used for a 45-day rearing trial. The pigs were blocked by their initial body weight and randomly assigned to one of two rearing systems: a conventional indoor rearing system (IN, *n* = 12; 6 castrated males and 6 females) and an outdoor rearing system (OUT, *n* = 12; 6 castrated males and 6 females). The indoor (IN) group was housed in a conventional facility equipped with fully slatted floors and a deep-pit manure-removal system. The IN animals were divided into two replicate pens (12 m^2^ per pen, 6 pigs/pen), providing a space allowance of 2.0 m^2^ per pig. The outdoor (OUT) group was reared in an open yard securely enclosed by fencing, characterized by a natural dirt floor with sparse grass cover. The OUT group was also divided into two replicate pens (50 m^2^ per pen, 6 pigs/pen), offering an expansive space allowance of approximately 8.3 m^2^ per animal. To protect the animals from solar radiation and adverse weather, each outdoor pen was equipped with a large semi-cylindrical metal shelter, which was sufficiently sized to accommodate all pigs within the pen simultaneously. Furthermore, water was provided via nipple drinkers installed beneath a separate wooden shade structure, ensuring the animals remained protected from direct sunlight while drinking. The 45-day feeding trial commenced in late September 2024. During the entire experimental period, both groups of pigs were maintained under similar general environmental conditions (temperature and humidity) and were fed the same standard commercial corn-soybean meal-based diet. Feed and water were provided ad libitum through standard feeders and nipple drinkers installed in the respective rearing environments. Detailed records of the week’s feed intake, daily temperature, and humidity for both the indoor and outdoor environments during the experimental period are provided in the Supplemental Materials (Figures S1–S3). In the indoor facility, the temperature ranged from 19.4 °C to 23.5 °C, and the relative humidity fluctuated between 23% and 91% (averaging approximately 53%). In the outdoor yard, the ambient temperature ranged from 20.0 °C to 23.7 °C, with the relative humidity ranging from 30% to 87% (averaging approximately 57%).

### 2.2. Growth Performance and Organ Index

The body weight (BW) of each individual pig and the feed consumption of the groups were recorded at the beginning (day 0) and the end of the experiment (day 45). These data were used to calculate the average daily gain (ADG) for the entire experimental period. At the conclusion of the feeding trial, the pigs were slaughtered in accordance with standard commercial protocols. The spleen of each pig was immediately isolated and weighed to determine the absolute organ weight. The spleen organ index was subsequently calculated as the ratio of the spleen weight to the final live body weight of the respective pig.

### 2.3. Blood Collection and Analysis

At the end of the 45-day experiment, fasting blood samples were collected from the jugular vein of the pigs prior to slaughter. For hematological profiling, whole blood was collected into Vacutainer tubes containing EDTA (Becton Dickinson, Franklin Lakes, NJ, USA). For serum biochemical analysis, blood was collected into serum separator tubes (SST) (Becton Dickinson, Franklin Lakes, NJ, USA). The serum tubes were centrifuged to separate the serum, which was then carefully transferred to plastic microtubes and stored appropriately until analysis. All hematological and serum biochemical analyses were performed by the NC State Veterinary Hospital Diagnostic Laboratory System (Raleigh, NC, USA). The complete blood count (CBC) parameters, including white blood cells (WBC), red blood cells (RBC), and platelet indices, were measured using an automated hematology analyzer (Siemens Healthineers, Cary, NC, USA). Serum samples were analyzed for protein and nitrogen metabolism (total protein, albumin, globulin, blood urea nitrogen, creatinine), energy and lipid metabolism (glucose, triglycerides), liver and muscle enzyme activities (AST, ALP, GGT, CK), and essential minerals and electrolytes (calcium, phosphorus, magnesium, sodium, potassium, chloride) using an automated clinical chemistry analyzer (Siemens Healthineers, Cary, NC, USA). Additionally, serum samples were screened for hemolysis and lipemia indices to ensure sample integrity, and all samples analyzed were within the acceptable range for analytical accuracy.

### 2.4. Statistical Analyses

All collected data, including growth performance, organ index, hematological, and serum biochemical parameters, were analyzed using an independent samples *t*-test to compare the differences between the indoor (IN) and outdoor (OUT) rearing systems. The statistical analysis was performed using appropriate statistical software (GraphPad Prism 10). The False Discovery Rate (FDR) correction using the Benjamini–Hochberg procedure. The FDR data were provided as Supplementary Material (Table S1). Data are expressed as means with the standard error of the mean (SEM). Differences were declared statistically significant at *p* < 0.05.

## 3. Results

### 3.1. Growth Performance

Body weight changes were recorded during the animal trial to show the growth performance, and the gain-to-feed ratio was calculated. The growth performance of those two groups is presented in Table 1. There are no significant differences between the IN and OUT groups in initial body weight, final body weight, or average daily gain (*p* > 0.05). The results indicate that outdoor rearing did not negatively affect the growth performance of the pigs.

### 3.2. Spleen Weight and Organ Index

**Figure 1:**
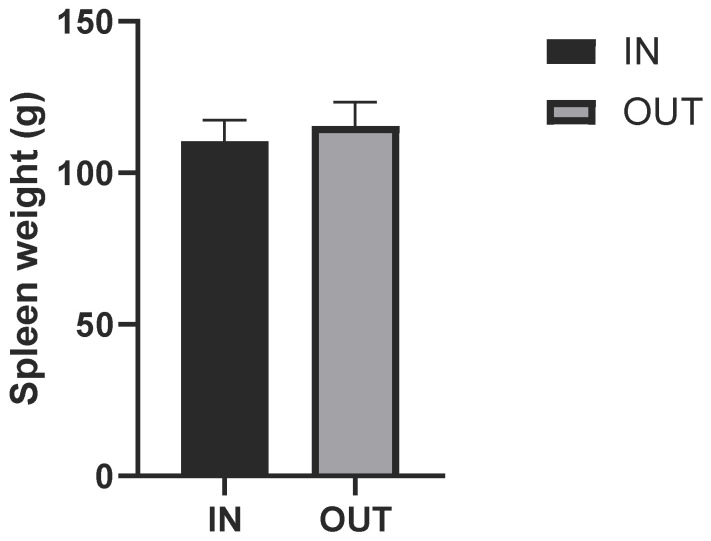
Spleen weight of indoor and outdoor Duroc pigs. Note: IN, indoor Group; OUT, outdoor group.

**Figure 2:**
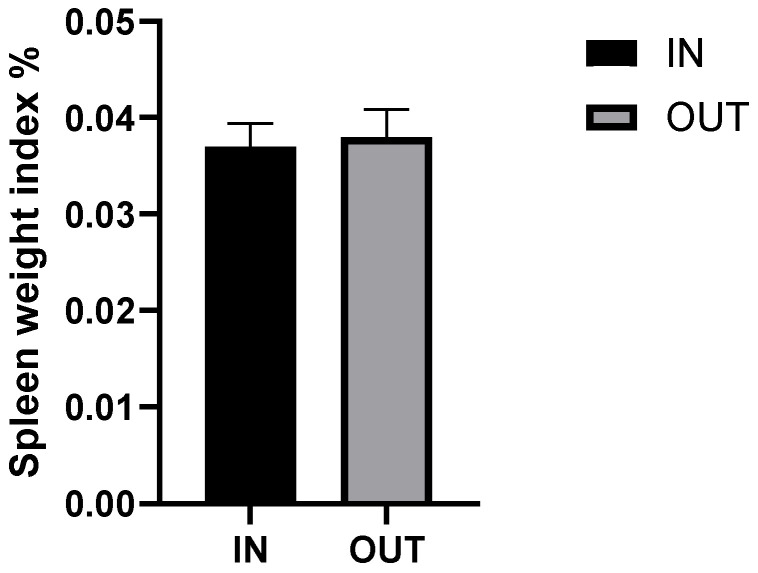
Spleen weight index of indoor and outdoor Duroc pigs. Note: IN, indoor Group; OUT, outdoor group; Spleen weight index % = Spleen weight/Final body weight.

The spleen weight of each pig was determined after slaughter. The spleen weight and the spleen weight to body weight data were shown in Figure 1 and Figure 2. There were no significant differences found in the weight of the spleen and the organ index between the IN and OUT groups (*p* > 0.05). Outdoor rearing did not affect spleen growth in Duroc pigs.

### 3.3. Hematological Parameters

The hematological profiles, including white blood cells (WBC), red blood cells (RBC), and platelets, are presented in Table 2, Table 3 and Table 4. Regarding the leukocyte profile (Table 2), the total WBC count and the counts of specific immune cells (neutrophils, lymphocytes, monocytes) did not differ between the groups (*p* > 0.05). Similarly, no significant differences were observed in erythrocyte indices (Table 3), such as RBC count, hemoglobin, and hematocrit (*p* > 0.05). For platelet parameters (Table 4), the plateletcrit (PCT) tended to decrease in the outdoor group compared to the indoor group (*p* = 0.08). However, the platelet count (PLT) and mean platelet volume (MPV) did not differ between treatments (*p* = 0.16 and *p* = 0.75, respectively).

### 3.4. Serum Biochemical Parameters

Serum biochemical parameters are presented in Table 5. The total protein concentration increased in the outdoor group compared to the indoor group (*p* = 0.04). However, globulin, albumin/globulin ratio, urea nitrogen (BUN), and creatinine concentrations did not differ between the groups (*p* > 0.05). Regarding energy and lipid metabolism, the triglyceride concentration increased significantly in the outdoor group (*p* = 0.02). For minerals and electrolytes, calcium and sodium concentrations were significantly higher in the outdoor group than in the indoor group (*p* < 0.01 for both). Magnesium concentration tended to increase in the outdoor group (*p* = 0.11). Other minerals and electrolytes, including phosphorus, potassium, and chloride, did not differ between the rearing systems (*p* > 0.05). Finally, the activities of liver and muscle enzymes, including aspartate aminotransferase (AST), alkaline phosphatase (ALP), gamma-glutamyl transferase (GGT), and creatine kinase (CK), did not differ between the indoor and outdoor groups (*p* > 0.05).

## 4. Discussion

Growth performance parameters in pigs, such as average daily gain (ADG) and gain-to-feed ratio, are direct indicators of swine production efficiency and are highly sensitive to environmental stressors [11]. Outdoor rearing typically increases physical activity and exposes pigs to fluctuating temperatures, which can increase maintenance energy requirements. If feed intake does not compensate for this increased energy expenditure, growth performance may be negatively affected [12]. In this study, the initial BW, final BW, and ADG did not differ between the IN and OUT groups. This indicates that the Duroc pigs were able to meet the thermoregulatory and physical demands of the outdoor environment without compromising their growth efficiency. According to our records, the temperature and humidity between the indoor and outdoor treatments were similar; the detailed records are provided in the Supplemental Materials. The effect of outdoor rearing on pig performance is a topic of debate among researchers. While some studies observed a reduction in ADG and feed efficiency due to increased energy loss for activity and thermoregulation [13], others, consistent with our results, reported that providing outdoor access had no negative impact on the overall growth performance of growing pigs and even increased the growth rate [14,15]. The lack of significant difference in our study suggests that the commercial diet provided sufficient energy and nutrients, and the Duroc breed possesses strong environmental adaptability to maintain optimal growth outdoors.

Although a complete growing-finishing cycle typically requires 90 to 120 days, the 45-day duration in this study was specifically selected. This timeframe effectively captures the critical window for physiological and metabolic acclimatization to the outdoor environment, while minimizing the confounding effects of long-term seasonal weather variations. The spleen is a primary peripheral lymphoid organ, and its relative weight (organ index) is commonly used as a macroscopic indicator of systemic immune response and overall health status [16,17]. Increased spleen weight (hypertrophy) typically occurs during chronic infection, severe environmental stress, or systemic inflammation as the body increases the production and proliferation of immune cells [18]. In this study, there were no significant differences found in the absolute weight of the spleen and the spleen organ index between the IN and OUT groups. This indicates that outdoor rearing did not induce chronic immune stress or systemic inflammation in the Duroc pigs. Higher total white blood cell (WBC) counts were observed in the OUT group, primarily associated with increased absolute counts of neutrophils and monocytes. Furthermore, serum enzymes such as creatine kinase (CK) and aspartate aminotransferase (AST) are widely utilized as biomarkers for muscle damage and liver stress [19,20]. Excessive physical exertion or environmental stressors can cause cellular membrane leakage, leading to elevated levels of these enzymes in the blood [21]. In the current study, the activities of AST, ALP, GGT, and CK did not differ between the rearing systems. Combined with the spleen data, these results suggest that although outdoor rearing exposes pigs to fluctuating environmental factors and increased physical activity, the pigs exhibited physiological adaptations within normal reference ranges, showing no evidence of pathological tissue damage or immune system overactivation.

Blood total protein and albumin levels reflect the nutritional status, hepatic protein synthesis capacity, and overall amino acid utilization of the animal [22]. An increase in feed efficiency or protein utilization typically results in elevated blood total protein levels [23]. In this study, the total protein concentration increased significantly, and albumin tended to increase in the outdoor group compared to the indoor group. This explains that despite the potentially higher energy expenditure required for thermoregulation and physical activity in the outdoor environment, the outdoor-reared pigs maintained an optimal or even enhanced protein metabolism. Since the growth performance (ADG) did not differ between the two groups, the elevated serum proteins suggest that outdoor rearing may stimulate protein turnover and nutrient transport efficiency. Previous studies have reported consistent results, indicating that moderate environmental stimulation and exercise can modulate blood circulation and amino acid utilization in skeletal muscle and liver tissues, allowing the pigs to fully compensate for the increased environmental demands [24,25].

Serum glucose and triglycerides are primary indicators of energy balance and lipid metabolism [26]. Outdoor rearing generally increases the physical activity of pigs, which necessitates a continuous and higher energy supply [27]. Increased energy expenditure promotes the mobilization of body fat reserves and hepatic glycogen [28]. This explains why, in this study, the triglyceride concentration increased significantly in the outdoor-reared pigs. The elevation of these metabolites suggests that the outdoor pigs actively upregulated their lipid mobilization and hepatic glucose output to meet the increased physiological demands of their environment. Consistent with these findings, previous studies have reported that free-range or outdoor pigs often exhibit altered lipid metabolism and enhanced energy substrate transport to support increased locomotion and thermogenesis without depleting their body reserves [29,30]. Serum calcium and sodium are strictly regulated electrolytes crucial for bone development, muscle contraction, and fluid homeostasis [31]. In the current study, calcium and sodium concentrations were significantly increased in the outdoor group. The higher serum calcium in outdoor pigs can likely be attributed to increased exposure to natural sunlight. Sunlight exposure promotes endogenous vitamin D3 synthesis in the skin, which subsequently enhances intestinal calcium absorption and bone mineralization [32]. Additionally, the elevated sodium and magnesium levels, while remaining within the normal physiological range, indicate that the outdoor pigs successfully maintained their osmotic balance and systemic hydration status despite increased respiration and activity outdoors. This robust homeostatic regulation further supports the hypothesis that Duroc pigs possess strong physiological adaptability to outdoor environments. It is crucial to note that although statistically significant differences were observed in certain hematological and serum biochemical parameters between the IN and OUT groups, the absolute values for all measured indices in both groups remained strictly within the established normal physiological reference intervals for healthy swine [33]. Therefore, these alterations do not indicate pathological stress or organ dysfunction. Instead, these modest modulations within the normal physiological range reflect a successful homeostatic adjustment and physiological acclimatization of the Duroc pigs to the outdoor rearing environment, including increased physical activity and exposure to natural elements. Future studies incorporating multi-omics approaches, such as transcriptomics and metabolomics, are recommended to elucidate the underlying molecular mechanisms and functional genes driving these physiological adaptations to the outdoor environment.

## 5. Conclusions

In conclusion, this study demonstrates that outdoor rearing does not compromise the growth performance or induce chronic physiological stress in Duroc pigs. The pigs exhibited physiological adaptability to the outdoor environment, characterized by variations in energy mobilization, protein turnover, and mineral homeostasis within standard physiological ranges. The absence of pathological tissue damage suggests that the Duroc breed possesses the capacity to adjust to varying environmental conditions. These findings indicate that outdoor rearing may be a viable production system that supports animal welfare while maintaining normal physiological status and production efficiency.

## Figures and Tables

**Table 1:** Effects of outdoor rearing on the growth performance and feed efficiency of Duroc pigs.

Parameters	IN		OUT		*p*-Value
	Mean	SEM	Mean	SEM	
Initial body weight (kg)	26.67	2.04	26.81	2.41	0.97
Final body weight (kg)	62.7	2.98	63.65	4.20	0.85
ADG (kg)	0.83	0.03	0.80	0.05	0.60

Note: IN, indoor Group; OUT, outdoor group.

**Table 2:** Effect of outdoor rearing system on leukocyte parameters and differential counts of Duroc pigs.

Item	IN		OUT		*p*-Value
	Mean	SEM	Mean	SEM	
White Blood Cells (WBC)					
Total Count (K/µL)	15.15	0.51	17.79	0.70	<0.01
Neutrophils (NEUT)					
Absolute Count (#, K/µL)	5.05	0.38	7.25	0.44	<0.01
Percentage (%)	32.99	1.80	40.68	1.77	<0.01
Monocytes (MONO)					
Absolute Count (#, K/µL)	0.85	0.05	1.11	0.07	<0.01
Percentage (%)	5.55	0.21	6.25	0.30	0.07
Eosinophils (EO)					
Absolute Count (#, K/µL)	0.41	0.04	0.79	0.11	<0.01
Percentage (%)	2.72	0.30	4.35	0.48	<0.01
Lymphocytes (LYMPH)					
Absolute Count (#, K/µL)	8.84	0.31	8.63	0.43	0.69
Percentage (%)	58.69	1.77	48.68	1.87	<0.01

Note: IN, indoor Group; OUT, outdoor group; #, number.

**Table 3:** Effect of outdoor rearing system on erythrocyte parameters and indices of Duroc pigs.

Item	IN		OUT		*p*-Value
	Mean	SEM	Mean	SEM	
Erythrocyte Indices					
Red Blood Cell (RBC) Count (M/µL)	7.50	0.11	7.38	0.34	0.34
Hemoglobin (HGB, g/dL)	13.17	0.17	12.93	0.08	0.19
Hematocrit (HCT, %)	42.63	0.59	43.04	0.35	0.54
Mean Corpuscular Volume (MCV, fL)	56.85	0.50	58.34	0.50	0.04
Mean Corpuscular Hemoglobin (MCH, pg)	17.58	0.17	17.55	0.13	0.90
Mean Corpuscular HGB Conc. (MCHC, g/dL)	30.91	0.16	30.05	0.18	<0.01
RDW-CV (%)	90.82	8.73	117.91	12.05	0.08
RDW-SD (fL)	41.69	0.59	42.63	0.69	0.30
Reticulocytes (RET)					
Absolute Count (#, K/µL)	90.92	8.73	117.91	12.05	0.09
Percentage (%)	1.21	0.41	1.61	0.17	0.07

Note: IN, indoor Group; OUT, outdoor group; RDW-CV: Red blood cell distribution width-coefficient of variation; RDW-SD: Red blood cell distribution width-standard deviation; #, number.

**Table 4:** Effect of outdoor rearing system on platelet parameters of Duroc pigs.

Item	IN		OUT		*p*-Value
	Mean	SEM	Mean	SEM	
Platelets (10^9^/L)	462.62	20.52	390.33	45.10	0.16
Mean platelet volume (MPV) (fL)	12.76	0.15	12.87	0.29	0.75
Plateletcrit (PCT) (%)	0.59	0.02	0.49	0.05	0.08

Note: IN, indoor Group; OUT, outdoor group.

**Table 5:** Effect of outdoor rearing system on serum biochemical parameters of Duroc pigs.

Item	IN		OUT		*p*-Value
	Mean	SEM	Mean	SEM	
Protein & Nitrogen Metabolism					
Total Protein (g/dL)	5.92	0.08	6.33	0.14	0.03
Albumin (g/dL)	4.16	0.05	4.3	0.05	0.08
Globulin (g/dL)	1.77	0.06	2.02	0.17	0.20
Albumin/Globulin Ratio (A/G)	2.37	0.10	2.23	0.18	0.48
Urea Nitrogen (BUN, mg/dL)	11.00	0.70	10.50	0.46	0.56
Creatinine (mg/dL)	1.05	0.07	1.06	0.06	0.90
Energy & Lipid Metabolism					
Glucose (mg/dL)	106.50	1.75	115.13	4.28	0.09
Triglycerides (mg/dL)	18.10	1.59	27.25	2.59	0.01
Enzyme Activity & Muscle/Liver Health					
Aspartate Aminotransferase (AST, U/L)	35.27	2.18	34.43	2.86	0.82
Alkaline Phosphatase (ALP, U/L)	176.18	8.12	189.25	8.86	0.29
Gamma-Glutamyl Transferase (GGT, U/L)	34.36	2.37	29.38	3.17	0.23
Creatine Kinase (CK, U/L)	1206.88	145.54	1330.57	137.14	0.55
Minerals & Electrolytes					
Calcium (mg/dL)	10.37	0.08	10.81	0.12	<0.01
Phosphorus (mg/dL)	8.48	0.16	8.76	0.34	0.48
Magnesium (mg/dL)	1.82	0.04	1.93	0.05	0.12
Sodium (mmol/L)	141.45	0.58	144.75	0.65	<0.01
Potassium (mmol/L)	5.34	0.16	5.14	0.05	0.26
Chloride (mmol/L)	99.45	0.69	100.13	0.52	0.45

Note: IN, indoor Group; OUT, outdoor group.

## Data Availability

Data are available within the manuscript.

## Supplementary Materials

The following supporting information can be downloaded at: https://www.mdpi.com/article/10.3390/ani16071040/s1, Figure S1: Weekly Temperature during animal trial; Figure S2: Weekly Humidity during animal trial; Figure S3: Weekly feed intake during animal trial; Table S1: False Discovery Rate (FDR) calculations.
