# Supplementary material for: Effects of Outdoor Rearing System on the Growth Performance and Blood Parameters of Duroc Pigs
Source: Animals (Basel). 2026 Mar 28;16(7):1040. doi: 10.3390/ani16071040 (PMC13071995; doi:10.3390/ani16071040)
Supplement: Supplementary file 1 [file animals-16-01040-s001.zip › Supplementary Figures S1-S3.pdf]

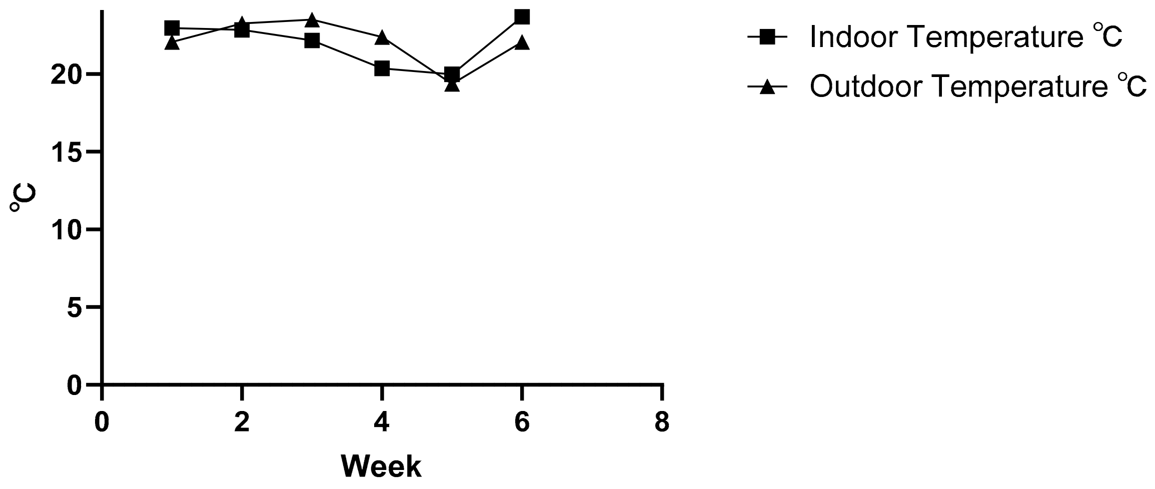

Figure S1: Weekly temperature during animal trial

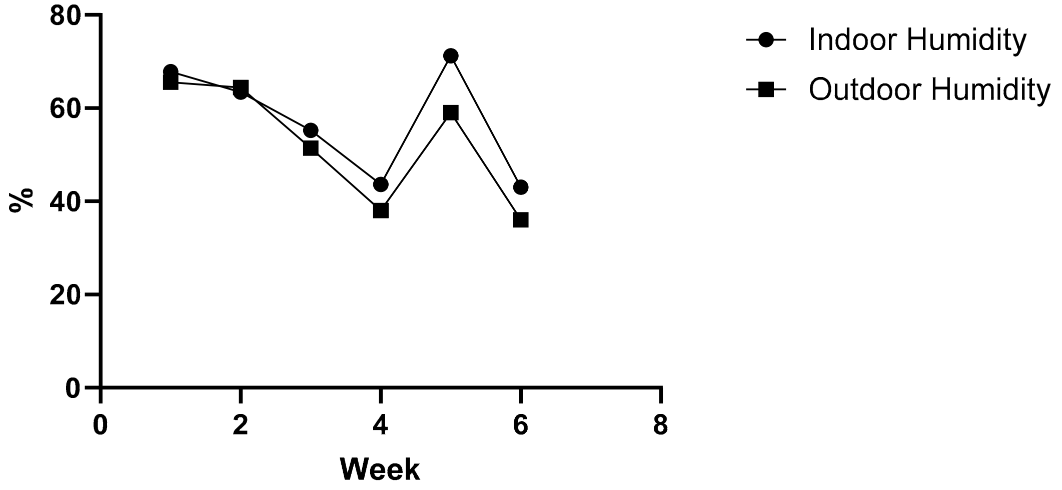

Figure S2: Weekly humidity during animal trial

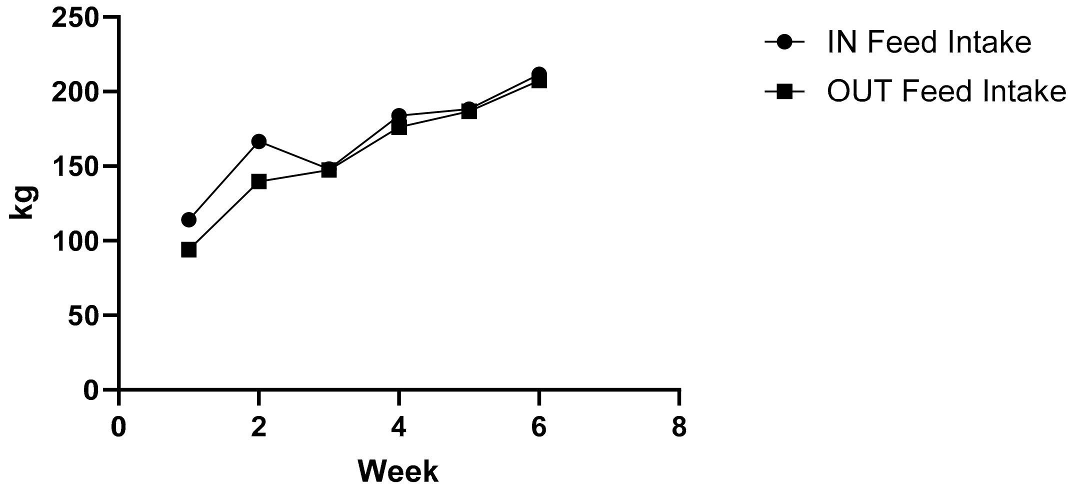

Figure S3. Weekly feed intake during animal trial
